# Finding the influential clinical traits that impact on the diagnosis of heart disease using statistical and machine-learning techniques

**DOI:** 10.1038/s41598-022-24633-4

**Published:** 2022-11-23

**Authors:** Iffat Ara Talin, Mahmudul Hasan Abid, Md. Al-Masrur Khan, Seong-Hoon Kee, Abdullah-Al Nahid

**Affiliations:** 1 Electronics and Communication Engineering Discipline, Khulna University, Khulna, 9208 Bangladesh; 2 Department of ICT integrated Ocean Smart Cities Engineering, Dong-A University, Busan, 49315 Korea

**Keywords:** Health care, Medical research

## Abstract

In recent years, the omnipresence of cardiac problems has been recognized as an epidemic. With the correct and quick diagnosis, both mortality and morbidity from cardiac disorders can be dramatically reduced. However, frequent medical check-ups are pricey and out of reach for a large number of people, particularly those living in low-income areas. In this paper, certain time-honored statistical techniques are used to determine the factors that lead to heart disease. Also, the findings were validated using various promising machine learning tools. Feature importance approach was employed to rank the clinical parameters of the patients based on the correlation of heart disease. In the case of statistical investigations, nonparametric tests such as the Mann Whitney U test and the Chi square test, as well as correlation analysis with Pearson correlation and Spearman Correlation were used. For additional validation, seven of the potential feature important based ML algorithms were applied. Moreover, Borda count was implemented to acknowledge the combined observation of those ML models. On top of that, SHAP value was calculated as a feature importance technique and for detailed evaluation. This research reveals two aspects of heart disease diagnosis.We found that eight clinical traits are sufficient to diagnose cardiac disorders, in which three traits are the most important sign of heart disease. One of the discoveries of this investigation uncovered chest pain, number of major blood vessels, thalassemia, age, maximum heart rate, cholesterol, oldpeak, and sex as sufficient clinical signs of individuals for the diagnosis of cardiac disorders. Over the above, considering the findings of all three approaches, chest pain, the number of major blood vessels, and thalassemia were identified as the prime factors of heart disease. The research also found, fasting blood sugar does not have a direct impact on cardiac disease. These findings will have the potency to be incredibly useful in clinical investigations as well as risk assessment for patients. Limiting the most critical features can have a significant impact on the diagnosis of heart disease and reduce the severity of health risks and death of patients.

## Introduction

Coronary heart disease, or simply heart disease, points out to a group of disorders that affects the heart. A report by World Health Organization (WHO) estimated that in the year 2019, heart disease cost about 17.9 million deaths worldwide, which mirrors 32$\%$ of total global death^1^. Miserably, about 75$\%$ of global deaths take place in lower to middle-income countries. According to a report by the Centers for Disease Control and Prevention (CDC), heart disease is the topmost health hazard in the United States^2^. A recent study by Hanif et al. discovered that in his country (Bangladesh), 27.5$\%$ of people in the age group 40–74 years are endangered to develop heart disease in the following 10 years^3^. A survey by the Bangladesh Bureau of Statistics (BBS) reported that 21.1$\%$ of entire mortality in the year 2020 was precipitated by heart attacks^4^. That manifests heart disease is the number-one life-threatening health condition in Bangladesh. Mortality and morbidity of patients can be brought down if heart disease can be diagnosed at its initial stage. Also, early detection of heart disease will allow patients to initiate necessary medical care and lifestyle modification.

Diagnosis of heart disease is many times complicated and costly. Multiple clinical examinations such as blood tests, an x-ray of the chest, ECG, holter monitoring, exercise stress tests, MRI scan, CT scan, coronary angiography, and suchlike many more are suggested by physicians to diagnose heart disease. Such a variety of clinical tests are not only expensive but also time-consuming. Some of the tests, like angiograms, can be uncomfortable as well as excruciating to patients. Analyzing clinical data of patients can provide vital clues in diagnosing a health condition. However, dealing with the clinical data of each patient manually can be time consuming and inefficient practice. Statistical analysis has vital functionality in medical science. The implementation of statistical philosophy in the biological study, especially in clinical and public health, is also known as biostatistics. Exploring historical statistics can be regarded as an essential factor in diagnosing, deciding to approach treatment, studying epidemiological events, and much more. Even so, handling such large data can be overwhelming if performed manually. Machine learning (ML)-based technologies are flourishing in the clinical field. Machine learning empowers practitioners to deal with a large number of clinical data that can be utilized to fabricate models which can play a part in reshaping the conventional health system. Many clinical parameters and symptoms of patients can indicate the presence of heart disease. Breathing difficulty and angina are the most prevailing indicator of heart disease. Hypertension, smoking habit, high levels of cholesterol, unhealthy weight, family history, age, etc., are a handful of the common factors that are guilty to contribute heart disease. But not all clinical features equally promote the risk of developing heart disease. Filtering out inessential and irrelevant clinical parameters can simplify the analytical process and computational cost. Feature selection intentionally removes less impactful and superfluous features from a dataset that results in better or at least similar accuracy. It is widely utilized to reduce the dimensionality of a dataset, and by which, computational complexity reduces to a great extent. The motive behind our analysis is to discover the appreciably prime features that can cause heart disease so that a reduced number of medical tests can put forward an optimum diagnosis of heart disease.

Researchers took several approaches to efficiently foresee as well as diagnose heart disease. When it comes to efficient diagnosis, dimensionality reduction can be a significant character. Many researchers favored a wide range of feature selection methods in addition to classification algorithms to conveniently anticipate heart disease. A research team led by MA. Jabbar performed multiple analyses regarding heart disease using a variety of ML tools. In 2015, they applied discretization together with multiple feature selection methods, including chi-square, One-R, Gain ratio, Relief as well as genetic search and finally used Naïve Bayes for classification^5^. Among the computational methods used in their study, a combination of One-R, Genetic Search, and Naïve Bayes could provide 86.29$\%$ accurate outcomes. In the next year, they made another effort to enhance the diagnosis process of heart disease. This time, they recommended another heart disease prediction model with Chi-Square and RF that achieved 83.70$\%$ accuracy^6^. Factors that lead men and women to heart disease were investigated by Nahar et al. by making use of three association rule generating algorithms those are- Apriori, Predictive Apriori and Tertius on the well-recognized Cleveland dataset by UCI^7^. They found women to be less likely to be invaded with heart disease. cp and exercise induced angina (exang) were found to be impactful enough to cause heart disease for both men and women. Tomar et al. developed a machine learning-based model that is able to diagnose the presence of heart disease^8^. They applied feature selection-based Least Square Twin Support Vector Machine (LSTSVM) on the Statlog dataset collected from UCI Machine Learning Repository to execute their work. Their analysis found 11 features to be significant over a total of 13 features and outperformed with the highest accuracy. Those 11 influential features are-age, chest pain, thalassemia, blood pressure, cholesterol, major vessels, the slope of the peak exercise ST segment, ECG, blood sugar, oldpeak and maximum heart rate. Another study published in 2017 by Yekkala et al. studied Statlog dataset for heart disease prediction by implementing three ML algorithms-AdaBoost, Random Forest and Bagged tree^9^. Moreover, they further investigated and sorted out the impactful clinical features with Particle Swarm Optimization (PSO). Among their proposed methods, PSO along with Bagged Tree performed incredibly well with 100$\%$ accuracy when analyzing only seven impactful features. Haq et al. proposed a hybrid ML model that can separate healthy individuals and heart disease patients based on certain health parameters^10^. Their team contrived seven popular ML classifiers that are-KNN, ANN, SVM, Logistic Regression, Naïve Bayes, Random Forest, and Decision tree, accompanied by K-fold cross-validation. As dimensionality reduction techniques, they availed LASSO, Relief, and mRMR. Logistic regression using 10-fold cross-validation coupled with Relief showed the highest 89$\%$ accuracy. In the same year, Khourdifi et al. published their study that combined a number of ML algorithms such as-SVM, KNN, Random Forest, Artificial Neural network, and Naïve Bayes with feature selection methods^11^. They implemented feature selection in two different approaches, Fast Correlation-Based Feature selection (FCBF) and Particle Swarm Optimization (PSO) based feature selection along with Ant Colony Optimization (ACO). Their analysis suggested two ML algorithms-KNN and RF with the most accurate outcome. Concerning accuracy, KNN and RF scored very close to each other, 99.65$\%$ and 99.6$\%$. Dubey et al. introduced multiple machine learning approaches to detect the presence of heart disease along with the ANOVA F-test. ANOVA F-test (AFS) was used to determine which of the clinical features of a patient contributes more to being affected with heart disease^12^. They utilized two popular datasets for heart disease commonly known as Cleverland and Statlog datasets from the UCI Machine Learning Repository. They classified the datasets both with and without performing feature selection with AFS. Classification along with AFS showed better accuracy. Seven clinical features namely cp, electrocardiographic results when resting (restecg), thalach, exang, oldpeak, slope of the peak exercise ST segment (slope), number of major blood vessels (ca), and thal were selected by the AFS method for improved performance. In an article by Garate-Escamilla et al., the researchers obtained impressive performance by using Chi square and principal component analysis (PCA) based feature selection along with Random Forest^13^. The accuracy that they obtained is given by- $98.7\%$ for Cleveland, $99.4\%$ for Cleveland-Hungarian (CH) and $99.0\%$ for Hungarian datasets. Singh et al. proposed a combination of multiscale wavelet packet (MSWP) transform, Fisher ranking method, Gaussian discriminant analysis (GDA), Linear Discriminant Analysis (LDA) along with extreme learning machine (ELM) that can diagnose coronary artery disease (CAD) with $100\%$ accuracy^14^. A literature by Anbarasi et al. showed that their model can accurately predict the most impactful features for heart diagnosis with less than half of primary features^15^. Their experiment concluded Decision Tree to be the best decision maker with $99.2 \%$ accurate outcomes. In 2020, Gupta et al. proposed a new model based on factor analysis of mixed data (FAMD) and Random Forest that acquired $93.44\%$ accuracy while analyzing Cleveland dataset^16^.

Our analysis focuses on figuring out the minimum number of clinical features that are significant in diagnosing heart disease. For this, we have not only employed ML-based algorithms but also took inspiration from traditional statistical methods. Also, we introduced a voting method in this study. Our analysis is organized into four major portions. In “Methods”, we have described our proposed methods along with a brief description of the used datasets and the classifiers. “Result and discussion” displays the result of our work. Here we also have summarized the analysis, the results, and the findings of our work. In “Conclusion”, we have concluded our work.

## Methods

**Figure 1:**
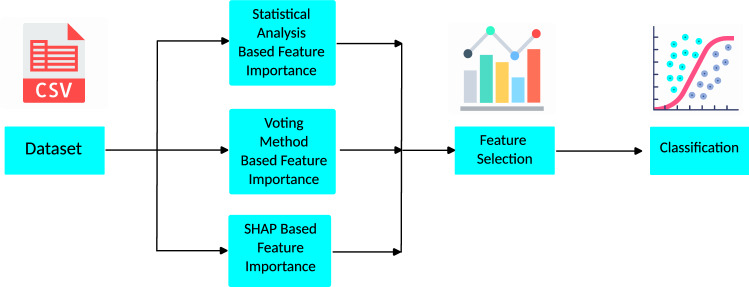
Outline of our study.

As stated in the previous section, this study aims to figure out the par-amount clinical traits of a patient that can contribute to the diagnosis of heart disease. To obtain this, we strategically split our work into two broad parts, feature selection, and classification. Feature selection figures out the foremost influential clinical features and classification portion diagnoses heart disease. The block diagram in Fig. 1 briefly illustrates our course of action.

For feature selection, the dataset that embodied the clinical history of patients was initially investigated in two different approaches. The first one is the traditional statistical analysis and the second one is machine learning-based feature importance analysis coupled with a voting method. Both ML-based feature importance methods and statistical analysis eliminated one feature as it was found to have no impact on the target variable. So, we omitted that feature for further analysis before anything else. Among the remaining features, two features received a mismatched opinion from statistical and ML-based methods in terms of importance. To steer clear of false-negative values, we have considered both two controversial features for further analysis. For classification, tree-based classifiers were employed.

### Description of the dataset

This study is based on a dataset donated in 1988 that is made up of combining four distinct databases: Cleveland, Hungary, Switzerland, and Long Beach V. This is one of the most used datasets by machine learning researchers for heart disease analysis till now^17^. It initially carried 76 features, but among these, 13 selected features are used for research all the time. So the dataset that we worked with deals with 13 clinical features of 1025 patients, and also holds a target variable that indicates the presence of heart disease. Table 1 delineates the dataset that is utilized for this study.

### Feature importance method

The feature importance method is a kind of approach to performing feature selection. Feature importance designates the features according to their usefulness for a certain classification. Feature importance can provide the foundation for feature selection. The least important features can be omitted for further analysis and thus can furnish the base of reducing dimensions. It holds a major portion of our analysis. We have implied a few well-known statistical methods to find out the correlation and association of features with the target variable. Also, to ensure proper investigation, we employed a few ML-based feature importance analyses along with a voting method.

**Table 1:** Description of the dataset.

Feature name	Feature specification	Categories	No heart disease	Heart disease	Total
Age	Age	24–39	15	42	57
		40–54	143	276	419
		55–69	327	188	515
		70–85	14	20	34
		Total	499	526	1025
Sex	Sex	Male	86	226	312
		Female	413	300	713
		Total	499	526	1025
cp	Chest pain	1 = Typical angina	375	122	497
		2 = Atypical angina	33	134	167
		3 = Non-anginal pain	65	219	284
		4 = Asymptomatic	26	51	77
		Total	499	526	1025
Trestbps	Resting blood pressure	90-119	84	117	201
		120–149	314	340	654
		150–179	87	66	153
		180–210	14	3	17
		Total	499	526	1025
chol	Cholesterol	120-130	182	244	426
		231–341	306	263	569
		342–453	11	16	27
		454–564	0	3	3
		Total	499	526	1025
fbs	Fasting blood sugar	<120mg/dl	417	455	872
		> 120 mg/dl	82	71	153
		Total	499	526	1025
Restecg	Electrocardiographic results when testing	0=Normal	283	214	Normal
		1 = ST-T wave abnormality	204	309	ST-T wave abnormality
		2 = LV hypertrophy	12	3	LV hypertrophy
		Total	499	526	1025
Thalach	Maximum heart rate	70-104	33	3	36
		105–139	189	74	263
		140–174	263	353	616
		175–2094	14	96	110
		Total	499	526	1025
Exang	Angina that is caused by anxiety	No	225	455	680
		274	71	71	345
		Total	499	526	1025
Oldpeak	ST depression due to exercise compared to rest	0–1.54	265	461	726
		1.55-3.00	167	59	226
		3.01–4.64	60	6	66
		4.65–6.2	7	0	7
		Total	499	526	1025
Slope	slope of the peak exercise ST segment	1 = Up sloping	46	28	74
		2 = Flat	324	158	482
		3 = Down sloping	129	340	469
		Total	499	526	1025
ca	Number of major blood vessels	1	160	66	226
		2	113	21	134
		3	60	9	69
		4	3	15	18
		Total	499	526	1025
Thal	Thalassemia	0	4	3	7
		Normal	43	21	64
		Fixed defect	132	412	544
		Reversable	320	90	410
		Total	499	526	1025

#### Feature importance with classical statistical analysis

We performed a handful of statistical analyses on the dataset, namely- Chi square test, Mann Whitney U-test, Karl Pearson Coefficient Correlation, Spearman Correlation. Chi Square test is a nonparametric hypothesis testing method. It is used to determine if there is any statistically significant relationship between expected values and observed values or not. It is used over and over by the researchers as a test of independence by utilizing a bivariate table. It is one of the most commonly used methods to determine if two variables are related or not. Mann Whitney U-test is a nonparametric test as well. This method is used to compare two sample means that are derived from the same population. It is implemented to determine if there is any statistically significant difference between the means or not. It is conveniently used when the data is non-normally distributed. Unlike the previous two tests, Pearson Coefficient Correlation measures the association between two variables. It is used to obtain linear associations between variables. The closer the value of the correlation coefficient is to 1, the more correlated the variables are. Spearman Correlation evaluates the association between variables as well, but unlike Karl Pearson Coefficient Correlation, it works with ranked values of variables instead of the raw primary data. While Pearson Correlation Coefficient measures linear relationship, Spearman correlation is used to express the monotonic association between variables.

#### Feature importance with voting method

Borda count is a rank-based voting method that is named after French mathematician Jean-Charles de Borda. Borda count is an advanced form of plurality method. In the plurality method, only the top preference of the population is considered. In Borda count, each balloter ranks the candidates according to their preference and assigns points to each choice. The first choice gets the highest point, and the last choice gets the least point. Scores given by all voters to candidates are summed up, and the candidate who gets the maximum point takes the crown. We have chosen seven classifiers that provide the rank of features, namely-Permutation with Random Forest, Permutation with CART, Permutation with KNN, Decision Tree, Random Forest, XGBoost, Permutation with XGBoost. Each feature selection method provided its own ranking of features. We decided to sum up all the outcomes of each feature selection method to estimate the most important features that cause heart disease. The outcomes of feature importance methods have been combined using Borda count.

#### SHAP value based feature importance

Shapley values or SHAP values are an idea that dates back to the 1950s and comes from the cooperative game theory literature. SHAP values were initially employed to fairly attribute a player’s contribution to a game’s eventual result. Assume we have a collaborative game in which a group of players plays together to achieve some value. SHAP values quantify the marginal donation of the each player to the ultimate result. If we consider our ML model as a game wherein various features“collaborate”to achieve an output (model prediction), we can assess the contribution of the each feature to the ultimate result.

### Used classifier

#### Classifiers for ranking features

Seven classifiers were chosen to rank the features with respect to their usefulness. Out of them, three were tree-based algorithms, and the remaining four models designated the features based on the permutation model. The classifiers that were used are-Decision Tree, Random Forest, XGBoost, Permutation with Random Forest, Permutation with CART, Permutation with KNN, and Permutation with XGBoost. Tree-based models rank the feature according to the decline of standard deviation and mean value of impurity. A major drawback of tree-based feature importance is that it can deceive the wrong ranking for the features with higher cardinal values. Permutation-based feature importance methods can be a wise alternative to overcome this problem. This method estimates the importance of a method by computing the prediction error of the model. For this, the permutation of a feature is performed to measure the reliance of the model on that feature. An increase in the model’s predictive error when altering the order of a feature indicates the importance of that feature. Correspondingly, unaffected performance represents the irrelevance of that feature and can be expunged.

#### Classifiers used for performance analysis

We have employed five well-known tree-based classifiers to obtain performance. These are- Random Forest, Gradient Boost, XGBoost, CatBoost, and LightBoost. All of these fall in the category of supervised learning models. Tree-based classifiers provide elevated performance with high stability, and also, they are pretty straightforward for result interpretation. These models can work with linear as well as non-linearly separable data. Our dataset contains clinical parameters of patients that have complex, non-linear relationships. Tree-based classifiers can be the best choice in such a case.

### Performance analysis

We divided our result and discussion section into two major parts. In the first place, we analyzed our given dataset with classical hypothesis testing and correlation methods. Moreover, we introduced ML-based feature importance methods so that we can compare the outcomes and come to a conclusion.

## Result and discussion

To obtain the most impactful features that may cause heart disease, we have taken two different approaches. The first one is the traditional bio-statistical analysis that employs not only two hypothesis testing methods but also two correlation methods. Our dataset embodies a good number of nominal and ordinal data, so we performed nonparametric hypothesis testing. As our dataset is non-normally distributed, we have implemented the Mann-Whitney U test and Chi-square test. Moreover, we employed ML-based feature importance methods along with a voting method.

### Feature importance with statistical analysis

#### Outcomes of hypothesis testing

As discussed before, here we have undertaken two hypothesis testing methods, Mann Whitney U test and Chi squared test. Table 2 shows the outcome of the tests.

**Table 2:** Mann Whitney U test and Chi squared test outcomes.

Feature name	Mann Whitney U test p value	Chi Squared test p value
Age	<0.001	<0.001
Sex	<0.001	<0.001
bp	<0.0017	<0.001
Trestbps	<0.001	<0.001
Chol	0.001	<0.001
fbs	0.188	0.188
Restecg	<0.001	<0.001
Thalach	<0.001	<0.001
Exang	<0.001	<0.001
Oldpeak	<0.001	<0.001
Slope	<0.001	<0.001
ca	<0.001	<0.001
Thal	<0.001	<0.001

**Table 3:** Pearson correlation outcomes.

Features	Value of r	Degree of correlation
cp	0.435	+ Moderate
Thalach	0.423	+ Moderate
Slope	0.346	+ Moderate
Restecg	0.134	No correlation
fbs	− 0.041	No correlation
Chol	− 0.1	No correlation
Trestbps	− 0.139	No correlation
Age	− 0.229	No correlation
Sex	− 0.28	No correlation
Thal	− 0.338	− Moderate
ca	− 0.382	− Moderate
Exang	− 0.438	− Moderate
Oldpeak	− 0.438	− Moderate

Not only Mann Whitney U-test, but also Chi Squared test computed the p value for fbs is 0.188 and for all the other features, the p-value is less than 0.001. That estimated with the exclusion of fbs, all other clinical factors showed a significant association with the target variable. As both Mann Whitney U-test and Chi Squared test ensured inconsequentiality of fbs, it may be considered the least impactful clinical feature contributing to heart disease.

#### Outcomes of correlation methods

##### Pearson correlation

 Pearson Correlation provided the correlation between each of the features and the target variable. Our investigation perceived that not only fbs, but also restecg, chol, trestbps, age, sex have no significant association with the target variable. cp, thalach, slope are the features that are positively correlated with the target variable. Whereas, thal, ca, exang, oldpeak are negatively correlated features. Table 3 shows the outcome of the Pearson Correlation.

##### Spearman correlation

In the case of Spearman Correlation, we get the exact same positively correlated features as we have obtained from the Pearson correlation. In the case of negatively correlated features, we obtained the same features but in a different order. Also, it is an important observation that Spearman correlation that this analysis identified fbs as one of the non-correlated features as the three previous analyses. The other non-correlated features are the same as Pearson Correlation, but with different correlation coefficients and different order as shown in Table 4.

**Table 4:** Spearman correlation outcomes.

Features	Value of r	Degree of correlation
cp	0.465	+ Moderate
Thalach	0.430	+ Moderate
Slope	0.369	+ Moderate
Restecg	0.147	No correlation
fbs	− 0.041	No correlation
Trestbps	− 0.115	No correlation
Chol	−0.133	No correlation
Age	− 0.240	No correlation
Sex	− 0.280	No correlation
Thal	− 0.399	− Moderate
Exang	− 0.438	− Moderate
Oldpeak	− 0.438	− Moderate
ca	− 0.453	− Moderate

**Figure 2:**
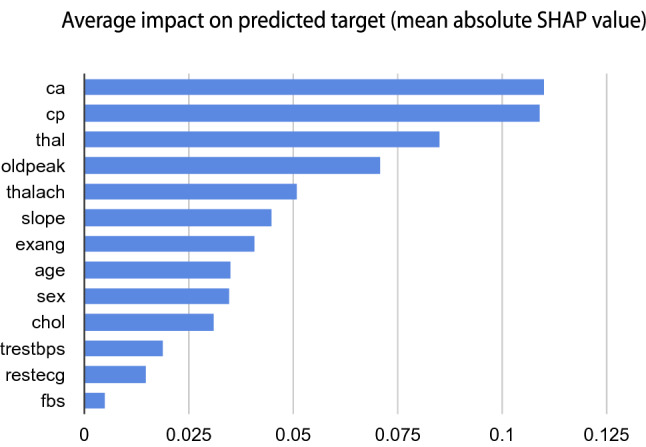
SHAP value based feature importance.

**Figure 3:**
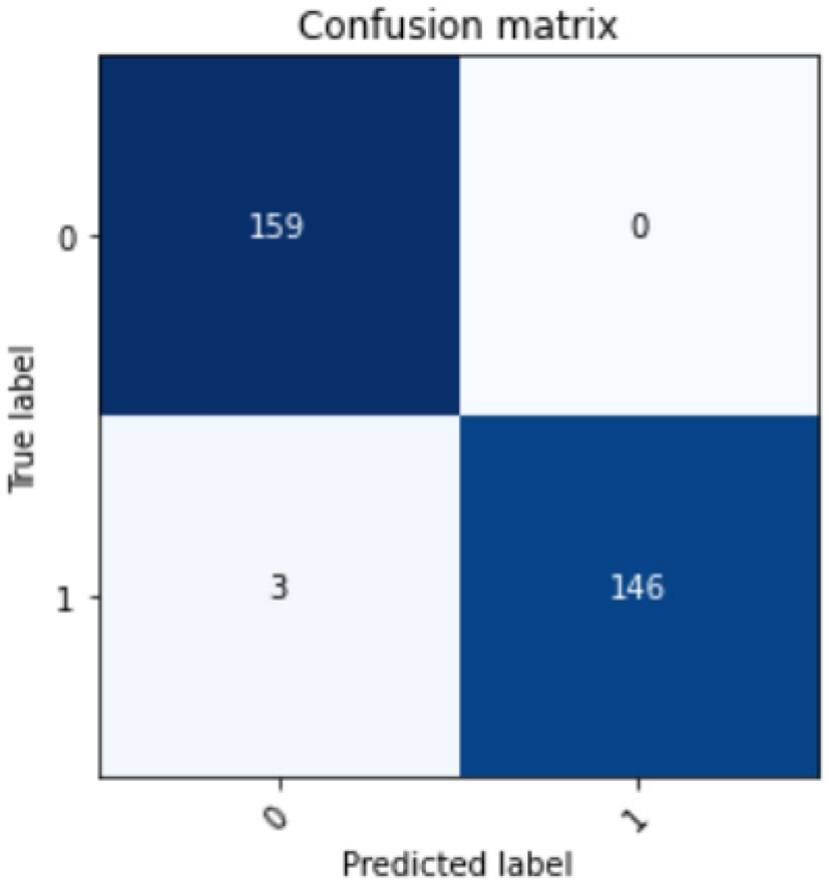
Confusion matrix with minimum numbers of features according to our analysis.

### Feature importance with machine learning models along with voting method

Aforesaid, to emphasize the validation of our analysis, ML-based feature importance was performed as well. Seven tree-based classifiers were employed that ranked the features and to amalgamate the findings, Borda count was utilized. SHAP value-based feature importance was exploited for enhanced inspection.

#### Feature importance with tree-based classifiers

Scores obtained by each feature importance algorithm concerning each clinical attributes are projected in Table 5. From Table 5, it can be clearly observed that each feature importance method came up with different scores for each clinical feature. As the scores of each feature did not correspond to each other, the ranking provided by algorithms would not be the same for each algorithm. To incorporate the ranks put forward by each feature importance algorithm, we implemented a democratic model, Borda count. In this analysis, Borda count treated the clinical features of the patients as candidates, and scores generated by feature importance methods are considered as votes. The outcomes of the Borda method are given below in Table 6.

**Table 5:** Importance of features of the dataset.

Feature name	Permutation with RF	Permutation with CART	Permutation with KNN	Decision tree	Random forest	XGBoost	Permuration with XGBoost
Age	0.006	0.119	0.097	0.081	0.092	0.043	0.043
Sex	0.008	0.082	0	0.027	0.044	0.059	0.054
cp	0.044	0.179	0	0.264	0.096	0.154	0.067
Trestbps	0.006	0.035	0.139	0.055	0.078	0.035	0.012
Chol	0.004	0.096	0.245	0.088	0.083	0.029	0.041
fbs	0	0.002	0	0.008	0.008	0.013	0
Restecg	0	0.018	0	0.009	0.018	0.036	0.008
Thalach	0.010	0.043	0.232	0.076	0.117	0.041	0.020
Exang	0.007	0.028	0	0.015	0.052	0.108	0.005
Oldpeak	0.015	0.090	0.004	0.070	0.114	0.104	0.071
Slope	0.002	0.018	0	0.019	0.043	0.077	0.002
ca	0.062	0.110	0.001	0.148	0.128	0.131	0.099
Thal	0.056	0.098	0	0.140	0.128	0.170	0.052

**Table 6:** Borda count outcome.

Feature name	Total counts	Order of features
cp	94	1
ca	80	2
Thal	69	3
Age	58	4
Thalach	57	5
Chol	50	6
Oldpeak	47	7
Sex	47	8
Exang	44	9
Trestbps	42	10
Slope	25	11
Restecg	19	12
fbs	12	13

A few crucial remarks can be made, cp had been calculated as the most influential factor, and fbs has been calculated as the least impactful at-tribute in Borda count likewise in statistical analysis. The mean absolute SHAP value of individual features was determined to support the Borda count results. The SHAP value of each clinical parameter of patients in relation to the target variable is depicted in Fig. 2. ca, cp, and thal were the top three Borda count features that scored extremely well in terms of SHAP value as well. As a logical consequence, cp, ca, and thal are by far the most responsible for heart disease.

As stated, Borda count offers a practicable designation of clinical features of potential heart disease patients that speaks for the importance of features. In spite of that, Borda count does not eliminate any feature like hypothesis testing methods. That implies it can not detect whether any feature is inessential, redundant or not. To obtain the indispensable features from the rank provided by Borda count, it was further analyzed with ML classifiers. As mentioned in the previous section, we implemented 5 tree-based ML classifiers, Random Forest, Gradient Boost, XGBoost, CatBoost, and LightBoost. We fed the classifier the features based on the ranking provided by Borda count to observe which features actually impact the performance most. The outcomes of the performance analysis are given in Table 7.

**Table 7:** Importance of features of the dataset.

Feature name	Light boost	CatBoost	RF	XGB	GB
Age	0.756	0.756	0.756	0.756	0.756
Sex	0.714	0.724	0.724	0.724	0.718
cp	0.821	0.828	0.821	0.821	0.818
Trestbps	0.903	0.896	0.925	0.851	0.857
Chol	0.971	0.942	0.971	0.867	0.877
fbs	0.971	0.955	0.971	0.916	0.916
Restecg	0.981	0.964	0.971	0.932	0.932
Thalach	0.981	0.981	0.99	0.925	0.935
Exang	0.990	0.981	0.99	0.938	0.955
Oldpeak	0.981	0.981	0.981	0.955	0.964
Slope	0.981	0.990	0.981	0.961	0.955
ca	0.990	0.990	0.981	0.948	0.955
Thal	0.990	0.990	0.990	0.968	0.951

**Table 8:** Classification report.

	Precision	Recall	f1-score	Support
No heart disease	0.98	1	0.99	159
Heart disease	1	0.98	0.99	149
Weighted avg	0.99	0.99	0.99	308

**Figure 4:**
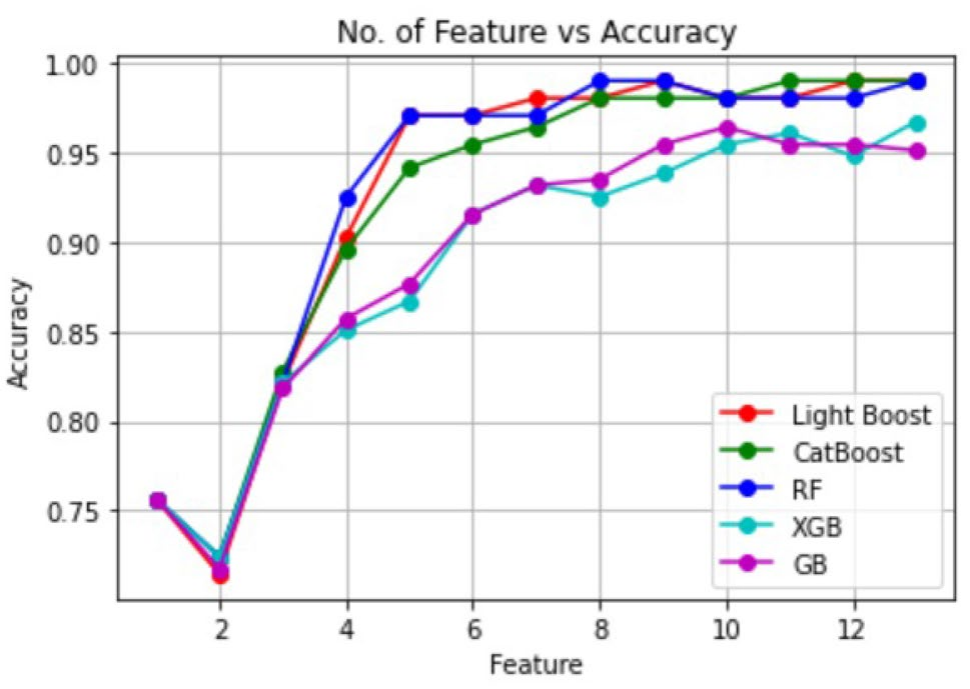
Classification result with different number of features (according to Borda Count).

Graphical representation can provide better insight into the analysis as given in Fig. 4. The above analysis represents how same accuracy can be obtained with a different set of features. Among five classifiers, Random Forest, CatBoost, and LightBoost performed with 99$\%$ accurate results when using all 13 features together. The remaining two classifiers, XGBoost and Gradient Boost, obtained 98 $\%$and 95$\%$ accuracy utilizing all 13 features. To investigate which of the classifiers may provide efficient performance with a lesser number of features, we fed the classifiers with the features based on the ranking provided by Borda count. While using the top 8 features suggested by Borda Count, Random forest obtained the exact same accuracy that with all 13 features. However, in the case of CatBoost and LightBoost, the accuracy was a bit lower, 98 $\%$with top 8 features. It is a clear observation that random Forest gave the most accurate outcome with less number of features. Here we performed a further analysis with Random Forest with those eight features namely- cp, ca, thal, age, thalach, chol, oldpeak, sex which are determined as the sufficient clinical parameters to diagnose heart disease. The confusion matrix, Fig. 3, summarizes the outcome and performance of Random Forest with suggested eight features.

It represents that our model successfully classified 159 true positive outcomes along with 146 true negative outcomes. When coming to misclassified values, it did not predict any false positive values but predicted only three false negative values, which is pretty impressive. Table 8 represents the detailed classification report with minimum numbers of features that performs similar to the raw dataset.For the absence of heart disease, the values of precision, recall, f1-score and support were rated as- 0.98, 1, 0.99, and 159, and for the presence of heart disease, these values were- 1, 0.98, 0.99 and 149. The weighted average value for precision, recall, and f1-score was obtained as 0.99 and the value of support was 308. The analysis result is excellent since the used dataset (Table 1) is a balanced dataset.

In the end, therefore, the findings of this work can be summarized as:

- cp, ca, thal, age, thalach, chol, oldpeak, sex are sufficient clinical trait to diagnose cardiac disorders.
- cp, ca and thal are the most important sign of heart disease.
- fbs does not have a direct impact on cardiac disease.

## Conclusion

The augmentation of heart disease throughout the global population has become an emerging concern for humankind. Heart disease takes away millions of lives annually. Spotting heart disease at an initial stage has the capability to redeem some of the sufferings of patients. Manual diagnosis of heart disease is not only time-consuming but also inefficient. Coupling ML with biostatistics makes it a versatile approach to dealing with increased and complex medical data of potential patients. For example, it is critical to understand what factors cause heart disease more than anything else so that healthcare providers may incorporate those results into their practice. To this end, many researchers have done their research works and declared 5-8 clinical traits of the patients as the prime factors to cause heart disease. The results of different groups are almost similar but not exactly the same. Our analysis also conjectured eight clinical traits of the patients of 13 clinical traits to be sufficient for heart disease diagnosis which are also not exactly the same as the previous research but similar. Also, this analysis explored cp, ca, and thal to be the most influential factors of heart disease. However, we emphasize our findings over others because we applied both the ML and statistical approach together, whereas previous works are done based on either the ML-based approach or statistical methods. In the future, other numerous heart disease datasets from different sources with more attributes could be explored to gain a more generalized result. Moreover, real-time data can be analyzed using the working learning model to verify that it is standardized and reliable through clinical correlation and validation.

## Data Availability

The data presented in this study are available on request from the corresponding author.
